# Clinical Outcomes of Early Antiviral Treatment for COVID-19 in a Japanese Long-Term Care Facility With Limited Resources: A Retrospective Study of 107 Residents

**DOI:** 10.7759/cureus.108521

**Published:** 2026-05-08

**Authors:** Norifumi Kudeken

**Affiliations:** 1 Internal Medicine, Kubagawa Medical Clinic, Naha, JPN

**Keywords:** antiviral agents, covid-19, frail elderly, long-term care, molnupiravir

## Abstract

Background

After COVID-19 was reclassified as a Class 5 infectious disease in Japan on May 8, 2023, long-term care facilities assumed primary responsibility for onsite management of infected residents. Detailed reports of clinical outcomes under this policy are scarce. SARS-CoV-2 infection remained clinically relevant despite repeated vaccination, including receipt of five or more vaccine doses in many residents, highlighting the need for early antiviral treatment in nursing home settings.

Methods

We conducted a retrospective observational study from May 8, 2023, to February 28, 2025, in a resource-limited Japanese long-term care facility. Among 113 residents diagnosed with COVID-19, six presented with moderate illness at diagnosis, operationally defined as decreased oxygen saturation with SpO₂ ≤ 93%, and were immediately referred to and admitted to the hospital. These residents were excluded from the primary analysis. The primary cohort comprised 107 residents with mild illness at diagnosis who received molnupiravir 800 mg twice daily for five days within eight hours of diagnosis. Outcomes included clinical progression, hospitalization after initiation of molnupiravir, mortality, and outbreak characteristics.

Results

Among the 107 treated residents, 104 were wheelchair-dependent, and three were bedridden. The mean age was 85.6 years, and 49 residents were aged ≥85 years. Prior COVID-19 vaccination was documented in 95 residents (88.7%). Eighty-three residents (77.5%) had documented receipt of at least five vaccine doses, whereas 24 residents (22.4%) had no documented receipt of at least five doses, including residents with zero to four recorded doses and those with unknown vaccination history. One resident (0.9%) deteriorated on day 3 despite early antiviral treatment, was hospitalized, and died after hospitalization. No in-facility deaths occurred. Six outbreak clusters were identified during the study period.

Conclusions

Early antiviral treatment for mild COVID-19 was feasible and associated with favorable outcomes in a resource-limited long-term care facility despite severe structural limitations, including multi-bedrooms and poor ventilation. These findings support rapid diagnosis, prompt molnupiravir initiation, and early hospital referral for residents with oxygen desaturation in frail older adults living in long-term care facilities.

## Introduction

COVID-19 outbreaks in long-term care facilities have been associated with disproportionately high morbidity and mortality among older adults with frailty and functional dependence [1-3]. In Japan, clustered outbreaks in elderly facilities have also highlighted the importance of facility structure, ventilation, and staffing constraints in outbreak control [4-6].

On May 8, 2023, COVID-19 was reclassified as a Class 5 infectious disease under Japanese law, shifting routine responsibility for onsite management toward long-term care facilities and community clinicians [7-9]. National surveillance reports have described outbreak frequencies in long-term care health facilities after this policy change [10,11], but peer-reviewed reports with resident-level clinical outcomes remain limited.

Breakthrough SARS-CoV-2 infections continued to occur despite repeated vaccination, including receipt of five or more vaccine doses in many residents, underscoring the need for early antiviral treatment strategies in nursing homes. We therefore examined the clinical outcomes of COVID-19 cases managed in a more than 50-year-old, resource-limited Japanese long-term care facility, focusing on early molnupiravir treatment, hospitalization, mortality, outbreak characteristics, and clinically relevant subgroup patterns.

## Materials and methods

Study design and setting

This retrospective observational study was conducted from May 8, 2023, to February 28, 2025, in a more than 50-year-old reinforced-concrete long-term care facility in Okinawa, Japan. The building had high airtightness, poor natural ventilation, and exclusively four bedrooms, except for one two-bed observation room. No private isolation rooms were available. Zoning was incomplete, and red and green zones shared ventilation lines.

Staffing consisted of one part-time physician and seven full-time nurses. Staff cohorting was not feasible, and 33 staff members contracted COVID-19 during the study period.

Participants and treatment

At diagnosis, residents underwent clinical assessment and pulse oximetry. Moderate illness requiring hospital referral was operationally defined as decreased oxygen saturation with SpO₂ ≤ 93%. Among 113 residents diagnosed with COVID-19, six met this criterion at diagnosis and were immediately referred to and admitted to the hospital without initiation of onsite antiviral treatment. These six residents were excluded from the primary analysis.

The primary analysis included 107 residents with mild COVID-19 at diagnosis who received molnupiravir 800 mg twice daily for five days, initiated within eight hours of diagnosis. No adverse effects attributable to molnupiravir were observed.

The standardized onsite management strategy focused on early antiviral treatment with molnupiravir, together with close clinical monitoring, hydration, antipyretics, and symptomatic care as needed. Non-antiviral adjunctive treatments, including nasal sprays, gargles, antibiotics, systemic corticosteroids, or multidrug outpatient protocols, were not routinely used as part of the facility protocol for residents with mild COVID-19. Antibiotics or systemic corticosteroids were reserved for cases with clinical suspicion of bacterial infection or progression to moderate or severe respiratory illness.

Outcomes and subgroup analyses

The primary outcomes in the treated cohort were clinical progression, hospitalization after initiation of molnupiravir, and mortality. Clinical progression was defined as deterioration after initiation of molnupiravir requiring hospital transfer or resulting in death. Baseline hospitalizations among residents with moderate illness at diagnosis were described separately and were not counted as progression events in the treated cohort.

Outbreak characteristics were also recorded. Exploratory subgroup analyses assessed progression according to age (≥85 vs. <85 years), documented vaccination status, diabetes, and mobility status. For vaccination status, residents were categorized as having documented receipt of at least five vaccine doses or having no documented receipt of at least five doses. The latter group included residents with zero to four recorded doses and those with unknown vaccination history.

Statistical analysis

Descriptive statistics were used to summarize resident characteristics, treatment patterns, and clinical outcomes. Continuous variables were summarized as means or medians, as appropriate, and categorical variables were summarized as counts and percentages. No formal hypothesis testing was performed because the study was descriptive and did not include a comparator group. All analyses were performed using Microsoft Excel (Microsoft® Corp., Redmond, WA).

Ethics

The study was conducted in accordance with the Declaration of Helsinki. The protocol was approved by the Kubagawa Medical Clinic Institutional Review Board (approval no. KM-2025-03, approved April 15, 2025). The requirement for individual informed consent was waived because this was a retrospective observational study using routinely collected clinical data.

## Results

Resident characteristics

The baseline characteristics of the 107 residents treated with molnupiravir are summarized in Table 1. The mean age was 85.6 years, and 49 residents (45.8%) were aged ≥85 years. Most residents were wheelchair-dependent (104/107, 97.2%), and three residents (2.8%) were bedridden. Diabetes was present in 13 residents (12.1%).

**Table 1:** Baseline characteristics of the 107 residents treated with molnupiravir Values are presented as number of residents or n/N (%), unless otherwise indicated. Vaccination status was classified as documented receipt of at least five vaccine doses or no documented receipt of at least five doses. The latter category included residents with zero to four recorded doses and those with unknown vaccination history.

Characteristic	Value
Total residents treated with molnupiravir	107
Mean age, years	85.6
Age ≥85 years	49/107 (45.8%)
Wheelchair-dependent	104/107 (97.2%)
Bedridden	3/107 (2.8%)
Diabetes	13/107 (12.1%)
Prior COVID-19 vaccination documented	95/107 (88.7%)
Documented receipt of ≥5 vaccine doses	83/107 (77.5%)
No documented receipt of ≥5 vaccine doses	24/107 (22.4%)

Prior COVID-19 vaccination was documented in 95 residents (88.7%). Among all 107 treated residents, 83 residents (77.5%) had documented receipt of at least five vaccine doses, whereas 24 residents (22.4%) had no documented receipt of at least five doses. The latter group included residents with zero to four recorded doses and those with unknown vaccination history.

Clinical outcomes and exploratory subgroup findings

Clinical outcomes and exploratory subgroup findings are summarized in Table 2. Among the 107 residents with mild illness at diagnosis who received molnupiravir, 106 residents (99.1%) recovered without clinical progression. One wheelchair-dependent resident with uncontrolled diabetes deteriorated on day 3 despite early antiviral treatment, was hospitalized, and died after hospitalization. No in-facility deaths occurred.

**Table 2:** Clinical outcomes and exploratory subgroup findings Values are presented as n/N (%). Clinical progression was defined as deterioration after initiation of molnupiravir requiring hospital transfer or resulting in death. Subgroup analyses were exploratory and should be interpreted with caution because of the small number of progression events.

Outcome or subgroup	Progression, n/N (%)
Overall treated cohort	1/107 (0.9%)
Age ≥85 years	1/49 (2.0%)
Age <85 years	0/58 (0%)
Documented receipt of ≥5 vaccine doses	0/83 (0%)
No documented receipt of ≥5 vaccine doses	1/24 (4.2%)
Diabetes	1/13 (7.7%)
No diabetes	0/94 (0%)
Bedridden	0/3 (0%)
Hospitalization after molnupiravir initiation	1/107 (0.9%)
Death after hospitalization	1/107 (0.9%)
In-facility death	0/107 (0%)

Hospitalization after molnupiravir initiation occurred in one resident (0.9%), and death after hospitalization also occurred in one resident (0.9%). No adverse effects attributable to molnupiravir were observed.

In exploratory subgroup analyses, the single case of clinical progression occurred in a resident aged ≥85 years, without documented receipt of at least five vaccine doses, and with diabetes. No progression was observed among residents aged <85 years, those with documented receipt of at least five vaccine doses, those without diabetes, or bedridden residents.

The six residents excluded from the primary analysis had decreased oxygen saturation with SpO₂ ≤ 93% at diagnosis, were classified as having moderate illness, and were hospitalized immediately without initiation of onsite antiviral treatment. All six were wheelchair-dependent. 

Outbreak profile

**Figure 1:**
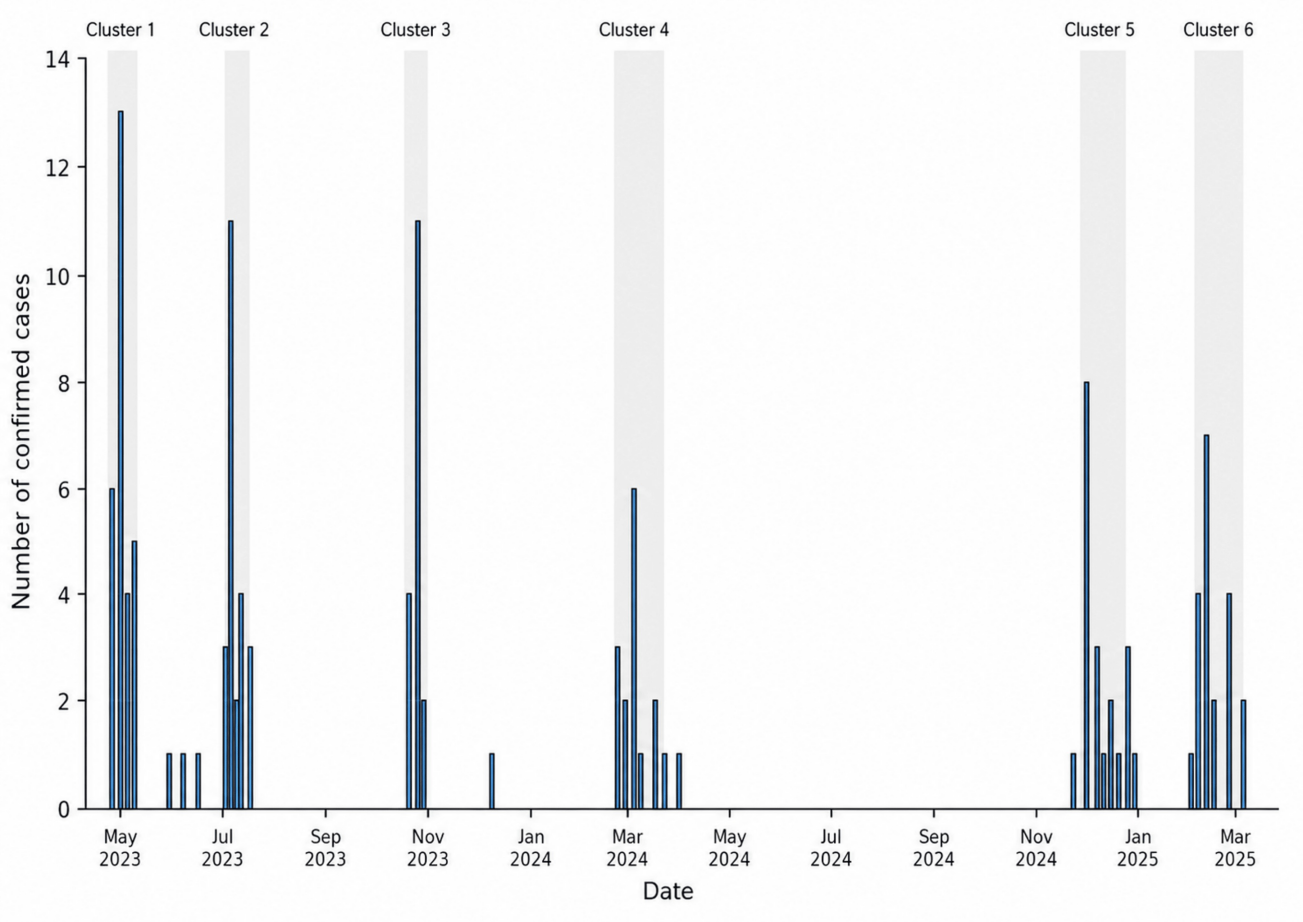
Epidemic curve of COVID-19 cases Daily number of confirmed COVID-19 cases during the study period from May 2023 to February 2025. Shaded areas indicate six outbreak clusters identified within the facility.

Figure 1 shows the daily number of confirmed COVID-19 cases during the study period from May 2023 to February 2025. Six outbreak clusters were identified during the study period. The clusters occurred on May 22-26, 2023; July 6-11, 2023; October 18-21, 2023; March 4-18, 2024; December 19-28, 2024; and February 3-13, 2025.

## Discussion

This study suggests that early antiviral treatment for mild COVID-19 can be implemented successfully in frail older adults living in a resource-limited long-term care facility, even when the physical environment is unfavorable for ideal infection control. Despite multi-bedrooms, incomplete zoning, and poor ventilation, only one of 107 residents treated promptly with molnupiravir deteriorated, and no in-facility deaths occurred.

The observed outcomes compare favorably with severe early outbreaks reported in long-term care facilities internationally [1-3] and are consistent with guideline recommendations and real-world studies showing the benefit of antiviral therapy among high-risk older adults and nursing home residents [12-23]. Previous real-world studies have evaluated antiviral agents such as molnupiravir, nirmatrelvir/ritonavir, and remdesivir for mild-to-moderate COVID-19. The present study specifically evaluated early molnupiravir-based management in a resource-limited long-term care facility. The single fatal case occurred in a wheelchair-dependent resident with uncontrolled diabetes, consistent with known risk factors for severe disease.

The occurrence of COVID-19 among residents who had received repeated vaccination, including five or more doses, indicates that vaccination alone may not eliminate outbreak-related clinical risk in frail nursing home populations. Previous observational data from the Cleveland Clinic have suggested that the number of prior COVID-19 vaccine doses may be associated with subsequent COVID-19 risk, although such findings should be interpreted cautiously because of the observational design and potential confounding [24]. In this context, early molnupiravir-based onsite management may serve as a practical adjunctive strategy to prevent clinical deterioration and reduce hospital transfers.

Several early outpatient COVID-19 treatment approaches have included multidrug or adjunctive strategies, such as antibiotics, corticosteroids, nasal or oral hygiene measures, and other supportive therapies [25-27]. However, the present study was not designed to evaluate such multidrug protocols. Instead, it focused on early antiviral-based onsite management, primarily using molnupiravir, in a severely infrastructure-limited long-term care facility. Therefore, our findings should be interpreted as real-world descriptive evidence supporting the feasibility and potential clinical value of prompt antiviral initiation and close monitoring in frail nursing home residents, rather than as definitive evidence of antiviral efficacy or evidence supporting broader multidrug treatment protocols.

The subgroup patterns should be interpreted cautiously because of the small number of progression events. Nevertheless, the findings are clinically plausible. Advanced age, absence of documented receipt of at least five vaccine doses, diabetes, and severe mobility dependence may identify residents who warrant particularly close monitoring. These findings are relevant in geriatric practice because rapid clinical decisions in long-term care settings are often made under structural and staffing constraints.

The study also has public health implications for aging societies in Asia. National reports in Japan have shown that outbreaks continue after Class 5 reclassification [10,11], and older facilities remain vulnerable because of shared rooms, poor ventilation, and staffing limitations. A workflow centered on rapid diagnosis, immediate antiviral access, and early hospital referral for residents with oxygen desaturation may reduce avoidable in-facility deterioration.

This study has several limitations. First, it was a single-center retrospective study conducted in one long-term care facility in Okinawa, which may limit the generalizability of the findings to other facilities with different staffing levels, infection-control resources, resident characteristics, or hospital-transfer policies. Second, the study did not include a non-treated control group; therefore, causal inference regarding the effectiveness of molnupiravir or other aspects of the management protocol is limited. Third, the findings should be interpreted as real-world descriptive outcomes of early antiviral-based onsite management rather than definitive evidence of antiviral efficacy. The number of clinical progression events was small, limiting subgroup inference. Residents with moderate illness at diagnosis, defined in this study by decreased oxygen saturation with SpO₂ ≤ 93%, were excluded from the primary analysis and hospitalized immediately; therefore, the findings primarily apply to residents with mild illness who were initially managed within the facility. Vaccination history was incomplete for some residents, and residents with unknown vaccination history were included in the group without documented receipt of at least five doses; therefore, vaccination subgroup findings should also be interpreted cautiously. Larger multicenter studies with comparator groups are needed to confirm these observations.

## Conclusions

Early antiviral treatment for mild COVID-19 was feasible in a resource-limited Japanese long-term care facility after Class 5 reclassification. In frail older residents, a practical strategy combining rapid diagnosis, prompt molnupiravir initiation, and timely hospital referral for residents with oxygen desaturation may help mitigate poor outcomes despite substantial structural constraints and repeated vaccination.
